# Assessment of testicular echotexture in young boars and its correlation with future semen production

**DOI:** 10.3389/fvets.2026.1828393

**Published:** 2026-05-06

**Authors:** Carolina L. A. Da Silva, Aston Bull, Lihandi van Niekerk, Kyla Ripley, Rodrigo M. Godinho

**Affiliations:** 1 Topigs Norsvin, ‘s-Hertogenbosch, Netherlands; 2 Topigs Norsvin South Africa, Pretoria, South Africa

**Keywords:** echotexture, semen quality, testicle, ultrasonography, young boars

## Abstract

Computer-assisted image analysis of testicular ultrasonography (US) can be used to assess testicular structure by objectively quantifying the brightness of pixels from US images [testicular echotexture]. In this study, testicular echotexture was assessed using B-mode testicular US in boars (*n* = 110) at 22, 24 and 26 weeks of age to obtain the echotexture traits such as: DKL (number of grey pixels) and Tubular Density [TUD, density of hypoechogenic areas, that is, seminiferous tubules (μm/cm^2^)]. Ejaculates (*n* = 2,498) were analysed for total number of sperm cells (TC, billion), motility and progressive motility 0 h (M0h, PM0h, %), and normal morphology (NM, %). LS means for US and semen traits were calculated per boar (Proc GLM, SAS), corrected for boar nested in line, weight (kg), age (weeks), and test month nested in season. Pearson’s correlation (Proc Corr, SAS) was used to assess the correlation between boar’s LS means (Least Square means) for US and semen traits. DKL was not significantly (*p* > 0.05) different in boars at 22 (158.1 ± 0.8), 24 (159.0 ± 0.7) and 26 weeks of age (158.9 ± 0.8), but TUD decreased (*p* < 0.05) from 22 weeks (106.0 ± 1.3) to 24 or 26 weeks (98.4 ± 1.0 and 98.1 ± 1.1 μm/cm^2^), showing an increase in testicular echogenicity with age, which happens during sexual development. DKL had a negative correlation (*p* < 0.05) with the TC (*R* = −0.54), M0h (*R* = −0.19), PM0h (*R* = −0.42), and NM (*R* = −0.40). TUD had a positive correlation (*p* < 0.05) with TC (*R* = 0.51) and with NM (*R* = 0.32). Thus, boars with higher tubular density produced a higher number of sperm cells with fewer morphological abnormalities, while boars with a higher number of grey pixels showed the opposite. Since DKL can be measured at 22 weeks of age, it could be the trait of choice for early selection of boars for productivity. This early selection could reduce the costs of semen production. Further research is needed to define the genetic basis of these traits, assess their relevance for breeding programs, and evaluate whether echotexture parameters can serve as objective markers of puberty onset in boars.

## 1 Introduction

The successful introduction of high genetic merit boars into semen production centers (SPC) depends on the approval of these boars in standard parameters of semen quality, such as the number of sperm cells per ejaculate and the percentage of these sperm cells that are motile and morphologically normal. For breeding companies and SPC, there are time related costs associated with this system. First, boars need to be old enough for semen collection, which will only occur after puberty has been reached in these animals, at 5 or even 6 months of age (30). Second, on farm level, puberty in boars is loosely defined as the onset of mounting behavior (1), but mounting behavior can be delayed or hindered by other factors such as stress, consequently delaying the capacity to collect semen from these animals (2). Third, there is high phenotypic variation of semen quality traits between ejaculates from the same boar, especially in young boars at start of reproductive life (3, 4). Moreover, phenotypic variation in semen quality traits can be related to semen sample handling before and during analysis. All together, these facts often make it necessary to assess multiple ejaculates before deciding on the usability of a boar. Consequently, when a boar is rejected for semen quality, mostly at 7 months of age or even later, several costs have already been incurred, including transport, veterinary tests, quarantine, feeding, and of stalling these boars. This is especially problematic when boars are transported to different countries. Due to all these facts, there is growing interest in non-invasive tools that could be used to assess a young boar’s potential for semen productivity and quality at earlier life, and also to objectively identify the onset of puberty.

Semen productivity and quality in post-pubertal boars are a consequence of a series of developmental changes happening in the testicles, from intra-uterine life until the onset of puberty (5). These changes include the growth of the seminiferous tubules leading to the initiation of spermatogenesis, with an expansion of luminal diameter and the appearance of sperm cells. As a result, testicular size increases during pubertal development in boars, up until 8 months of age, when testicular development reportedly ends (5). Due to the relationship between the development of seminiferous tubules, where sperm production takes place, and the growth of the testicles, boars with larger testicular size can produce a higher number of sperm cells per ejaculate. Consequently, selection of boars with larger testicles could be used to select boars with higher productivity per ejaculate (5, 6). Huang and

Johnson (6) reported that boars selected at 150 days of age for higher testicular size, showed higher daily sperm production and higher total number of sperm cells per ejaculate up to 450 days of life. However, testicular size does not correlate with other quality parameters of the ejaculates, and it is difficult to measure in non-pendular testicles such as the boars, without the use of calipers operated by experienced technicians (6) or, testicular ultrasonography (5, 7).

Ultrasonography has been used to measure testicular size in boars (5, 8), and the health of the testicular parenchyma as part of the breeding soundness evaluation of these animals (7, 9, 32). Recently, testicular ultrasonography in conjunction with computer-assisted image analysis has emerged as a potential non-invasive tool to predict ejaculate productivity and quality (10, 11). In this procedure, the grey-scale appearance of the ultrasonogram, also called testicular echotexture, is objectively quantified based on brightness or intensity of pixels, providing indications of microscopic structural changes in the developing testicles (10, 12). Thus, the objective of this study was to investigate the use of testicular ultrasonography and the computer-assisted image analysis of testicular echotexture in young pre-pubertal boars as a tool to predict their future ejaculate productivity and quality.

## 2 Materials and methods

### 2.1 Animals and study design

The study was conducted on a production nucleus farm in Mpumalanga, South Africa. Boars (*n* = 110) of four different Topigs Norsvin genetic lines (A, *n* = 75; B, *n* = 13; C, *n* = 12; and D, *n* = 10), selected at 22 weeks (±154d) based on genetic value during test, were used for this study. The genetic lines included Landrace, Large White, Pietrain and a Synthetic Line.

The boars were reared under test conditions from 9.5 weeks (±66d) to 22 weeks (±154d). From selection at 22 weeks (±154d), boars were relocated from the testing barns to the boar barn, where each boar was penned individually and fed on average 2.6 kg of a special diet for young boar’s once per day (NE = 9.4 MJ/kg; SID Lysine = 7.8 g/kg; crude protein = 140 g/kg). At 27 weeks (±189d), after selection, the boars were relocated from the nucleus barn to one of two semen collection centers situated in Mpumalanga (*n* = 43) and Gauteng (*n* = 57). Here, the boars were penned individually and were fed on average 3.0 kg of a specific boar diet once per day (NE = 9.4 MJ/kg; SID Lysine = 6.8 g/kg; crude protein = 140 g/kg). Water was *ad libitum* available for the boars.

### 2.2 Testicular ultrasonography and echotexture traits

Testicular echotexture was assessed via B- mode (Brightness-mode) ultrasonography of left and right testicles, with an Exapad Mini and a 7.5 MHz linear probe, with 50 mm depth and 25 mm focus (IMV Imaging®, IMV, France). Ultrasonography was done in boars at 22 weeks (±154d), 24 weeks (±168d) and at 26 weeks (±182d) of age. Prior to the ultrasound, boars were moved into a waiting area and both testicles were gently washed using wet cloth. Boars were then moved to a scale-crate with an open back door. Food was used as distraction. Prior to the ultrasonography, testicles were smeared with a clear ultrasound gel, and the probe was placed perpendicularly to the testicles, at the center of the testicular length, until the mediastinum could be completely visualized in the image. Two 4-s long ultrasound clips, or cineloops, were recorded per testicle. A total of four cineloops per boar at each age, 22, 24 and 26 weeks were recorded. On average, 8 to 10 boars were scanned per week, at different ages, over a period of 8 months. Every boar (*n* = 110) was scanned every 2 weeks. After each scanning session, the cineloops were analyzed using Ecotext software version 2.0 (Humeco®, Spain). The mediastinum was used as a reference point when determining the region of interest (ROI) for echotexture analysis. The Ecotext software evaluates each pixel in the image obtained that reflects the testicular parenchyma, whose intensity can be measured according to different shades of grey, black and white. Ecotext provides several echotexture traits (EC). In this study, two EC were used at normal resolution (1 and 2 below) and three at high resolution (3, 4 and 5) which are related to the anechoic areas, or the presence of fluid in the testicular parenchyma:

1. Number of grey pixels, the median of the mean grey values along all frames of the ROI.
2. DKL: measured as the median of the maximum grey pixels values for all frames of the ROI
3. Ecotext tubular density (μm/cm^2^) the density of anechoic areas,
4. Ecotext tubular area (%): total percentage of anechoic area,
5. Ecotext tubular diameter (μm): mean diameter of anechoic areas

### 2.3 Ejaculate collection and semen traits

Boars were relocated to the semen collection centers at 27 weeks of age, for semen production. Ejaculates (*n* = 2,498) were collected with an average collection interval of 4.9 ± 2.1 days, using a locally manufactured semi-automatic collection system, into filtered semen collection bags. Up to 5 min after collection, ejaculates were prediluted 1:1 (v:v) with commercial extender for long term semen storage at 17 °C (5 to 7 days). Once diluted, the ejaculates were cooled down to 22 °C before transport in temperature controlled boxes (±1.5 h’ drive) to the central lab for analysis.

Upon arrival, pre-diluted ejaculates were checked for volume with a monthly calibrated weighing scale. Samples from each ejaculate were prepared by diluting 250 μL of pre-diluted ejaculate into 2.25 mL of extender (1:9 dilution rate). These samples were heated at 38 °C in heating blocks for 5 to10 minutes prior to analysis for quality and concentration. Analysis was completed using an IVOS II (Hamilton Thorne, IMV, France) CASA system, with fixed volume chambered slides (Leja slides, 4 chambers, 20 μL, IMV, France), for motility (%), progressive motility (%), normal morphology (%), coiled tail (%), bent tail (%), distal midpiece reflex (DMR, %), proximal droplets (%), distal droplets (%) and concentration (million sperm cells per mL). Ejaculates approved in quality (>70% motility and normal morphology), were finally diluted to commercial artificial insemination doses, with 1.8 billion total sperm cells in 80 mL blisters. From the final diluted ejaculates, 1.5 mL samples were taken and kept in temperature-controlled cooling cabinets set to 17 °C for 96 h (4 days storage). After 96 h of storage, those samples were activated in heated blocks at 38 °C for 15 min and analyzed with the IVOS II as described for fresh (0 h) samples.

The following semen quality traits were used in this study: ejaculate volume (VOL, mL), sperm cell concentration (CON, million/mL), total concentration (TC, billion sperm cells per ejaculate), motility (M0h, M96h, %), progressive motility (PM0h, PM96h, %), normal morphology fresh (NM0h, %), and the percentages of individual abnormalities in fresh ejaculates: coiled tail (CT0h, %), Bent tail (BT0h, %), Distal Midpiece Reflex (DMR0h, %), proximal droplets (PD0h, %), distal droplets (DD0h, %).

### 2.4 Statistical analysis

Statistical analyses were performed using SAS Institute (Statistical Analysis Software, SAS®) software. Normality tests were performed using proc. univariate and averages for all variables were determined using proc. means. A general linear model (Proc GLM, SAS) was used to estimate least square means for each trait per boar as corrected boar values (CBV) for EC and for semen traits assuming the statistical models described below.

For EC, the model (model 1 below) included the fixed class effect of the individual boar nested in genetic line, testicle in which the examination was performed (left and right), week of scanning (22, 24 and 26 weeks) and the seasonality estimated as the month of the testicular ultrasound assessment nested in the season, plus the linear effect of weight (kg) of the boars at the moment of the testicular ultrasound. For semen traits (model 2 below), the model included the linear effect of the interval between semen collections (days) and the age of the boar at the moment of semen collection (months), the fixed class effect of the individual boar nested in genetic line, the seasonality estimated as the month of semen collection nested in the season. For both models, only significant (*p* < 0.05) CBVs were included in further analysis.

Model 1:

*f (EC traits) = weight_i_ (kg) + line_ii_(boar) + week of scanning_iii_ + testicle_iv_ + season(month)_v;_*

*i (82.0 kg to 174 kg).*

*ii [Genetic Lines A, B, C & D (110 boars)].*

*iii (22, 24 & 26 weeks).*

*iv (right & left).*

*v summer (Dec, Jan, Feb), Autumn (Mar), Winter (Aug) & Spring (Sept, Oct).*

Model 2:

*f (Semen traits) = collection Interval(d)_i_ + age (m)_ii_ + line_iii_(boar)_iii+_ season(month)_iv;_*

*i (3 to 15 days).*

*ii (5.8 to 14.4 m).*

*iii [Genetic Lines A, B, C & D (110 boars)].*

*iv summer (Dec, Jan, Feb), Autumn (Mar), Winter (Aug) & Spring (Sept, Oct).*

Finally, Pearson correlation coefficients (Proc Cor, SAS) were calculated between the statistically significant CBVs for EC and semen traits.

## 3 Results

Summary statistics for echotexture traits (EC) are presented in Table 1. Boars were on average 166.3 ± 11.5 days, or 5.5 months, of age when assessed for testicular echotexture via ultrasonography. The average number of grey pixels and DKL were 120.2 ± 9.3 and 159.5 ± 9.9, respectively, with DKL slightly higher than that of the number of grey pixels, as expected. The average tubular density in this boar population was 99.6 ± 15.6 μm/cm^2^ of testicular parenchyma, with an average tubular area of 2.0 ± 0.8% and tubular diameter of 62.8 ± 5.5 μm.

**Table 1:** Summary statistics for echotexture traits in boars at 22, 24, and 26 weeks of age.

Echotexture traits	*n*	Average	SD	Minimum	Maximum
Number of grey pixels	1,304	120.2	9.3	85.0	146.6
DKL	1,304	159.5	9.9	123.0	188.0
Tubular density (μm/cm^2^)	1,304	99.6	15.6	63.8	170.3
Tubular area (%)	1,304	2.0	0.8	0.8	7.5
Tubular diameter (μm)	1,304	62.8	5.5	50.8	94.0

For EC, there was no significant effect of boar weight (*p* > 0.05, Table 2). There was a significant effect of the boar nested in the genetic line on the EC (*p* < 0.05, Table 2). EC were significantly different between left and right testicles, age at ultrasonography and between different months and season of the year (*p* < 0.05, Table 2). Higher tubular density, area and diameter, and lower number of grey pixels and DKL were observed in the left testicles in comparison with the right testicles. Thus, right testicles have higher EC, with lower presence of productive parenchyma than the left testicles in young boars.

**Table 2:** Corrected boar values (CBVs) for echotexture traits in boars at 22, 24 and 26 weeks of age, in left and right testicles.

Echotexture traits	Age of ultrasonography (weeks)			Testicle		*p*-values				
	22	24	26	Left	Right	Line (boar)	Age (weeks)	Weight (kg)	Testicle	Season (month)
Number of grey pixels	117.6 (0.5)^a^	120.5 (0.3)^b^	121.7 (0.6)^b^	118.7 (0.2)^a^	120.8 (0.2)^b^	<0.0001	<0.0001	0.66	<0.0001	<0.0001
DKL	158.1 (0.8)	159.3 (0.7)	159.8 (0.6)	158.1 (0.3)^a^	160.0 (0.3)^b^	<0.0001	0.17	0.48	<0.0001	<0.0001
Tubular density (μm/cm^2^)	106.3 (0.9)^a^	98.9 (0.6)^b^	96.1 (1.0)^c^	102.4 (0.4)^a^	98.5 (0.4)^b^	<0.0001	<0.0001	0.42	<0.0001	<0.0001
Tubular area (%)	2.3 (0.1)^a^	2.0 (0.0)^b^	1.9 (0.1)^b^	2.2 (0.0)^a^	2.0 (0.0)^b^	<0.0001	<0.0001	0.14	<0.0001	<0.0001
Tubular diameter (μm)	64.7 (0.4)^a^	62.7 (0.2)^b^	61.8 (0.4)^c^	63.6 (0.2)^a^	62.5 (0.2)^b^	<0.0001	<0.0001	0.18	<0.0001	<0.0001

^a,b,c^Letters across different columns show statistical significant differences for age of ultrasonography examination and for left and right testicle (*p* < 0.05).

Boars at 22 weeks of age had a lower number of grey pixels (*p* < 0.05, Table 2) in comparison with boars at 24 and 26 weeks (117.6 ± 0.5 vs. 120.5 ± 0.3 and 121.7 ± 0.6, respectively). On the other hand, tubular density, area and diameter were significantly higher (*p* < 0.05) for boars at 22 weeks (106.3 ± 0.9 μm/cm^2^, 2.3 ± 0.1% and 64.7 ± 0.4 μm, respectively), than at 24 (98.9 ± 0.6 μm/cm^2^, 2.0 ± 0.0% and 62.7 ± 0.2 μm, respectively) and 26 weeks (96.1 ± 1.0 μm/cm^2^, 1.9 ± 0.1% and 61.8 ± 0.4 μm, respectively) of age. The only exception was DKL, whose values were did not differ (*p > 0.05*) in boars at 22, 24 and 26 weeks of age. So, DKL can be measured as early as 22 weeks of age in boars, despite the increase in testicular echogenicity observed from 22 to 26 weeks of age.

Finally, there was a decrease in the number of grey pixels (*p* < 0.0001) and DKL and an increase in tubular density, area and diameter from winter to summer, indicating a seasonality effect on EC.

All CBVs for EC were significant (*p* < 0.05), and consequently EC data from all boars (*n* = 110) were used for further analysis. Summary statistics for the CBVs for the EC traits are shown in Table 3.

**Table 3:** Summary statistics for the corrected boar values (CBVs) for echotexture traits in boars.

Echotexture traits	*n*	Average	SD	Minimum	Maximum
Number of grey pixels	110	119.8	10.3	99.7	139.5
DKL	110	159.1	11.4	134.6	181.2
Tubular density (μm/cm^2^)	110	100.4	16.4	67.6	134.0
Tubular area (%)	110	2.1	0.8	0.6	3.8
Tubular diameter (μm)	110	63.0	5.6	51.9	75.2

Summary statistics for semen traits are shown in Table 4. From 110 boars, 2.498 ejaculates were collected and used in this study. The average age of boars at semen collection was 9.1 ± 1.8 months, with collections happening on average every 5.2 ± 2.0 days. Average motility was 82.1 ± 11.0% immediately after collection, and 85.6 ± 13.7% after 96 h of storage. Total concentration was 54.4 ± 29.9 billion sperm cells per ejaculate.

**Table 4:** Summary statistics for semen traits, age at semen collection and interval between collections.

Boar and semen traits	*n*	Average	SD	Minimum	Maximum
Motility (%)	2,498	82.1	11.0	0.0	97.2
Progressive motility (%)	2,498	67.8	12.4	0.0	93.5
Morphology normal (%)	2,498	80.0	10.3	26.0	100.0
Bent tail (%)	2,498	2.1	2.9	0.0	66.7
Coiled tail (%)	2,498	0.2	0.9	0.0	25.0
DMR (%)	2,498	3.5	3.5	0.0	31.9
Proximal droplets (%)	2,498	8.2	5.5	0.0	66.0
Distal droplets (%)	2,498	6.5	4.0	0.0	41.9
Concentration (10^6^/mL)	2,498	154.3	69.8	0.0	520.0
Total concentration (billion)	2,498	54.4	29.9	0.0	220.3
Ejaculate volume (mL)	2,498	373.6	166.3	38.0	1258.0
Motility 96 h (%)	2,498	85.6	13.7	0.0	100.0
Progressive motility 96 h (%)	2,498	65.7	14.0	0.0	93.0
Age at collection (months)	2,498	9.1	1.8	5.8	14.4
Interval of collections (days)	2,498	5.2	2.0	3.0	15.0

Summary statistics for CBVs for semen traits are shown in Table 5. There was a significant effect of the boar nested in the genetic line for semen traits (*p < 0.05*, *p* values are in Supplementary Table 1 in Annex). CT0h, BT0h, PD0h, DD0h, VOL, CONC, and TC were significantly affected by the collection interval. PM0h, PD0h and VOL were significantly affected by the age of boars at collection. VOL, CONC and TC were significantly affected by season of semen collection, and so were NM0h, BT0h, DMR, PD0h and DD0h, M96h and PM96h.

**Table 5:** Summary statistics for the corrected boar values (CBVs) for semen traits in boars.

Semen traits*	*n*	Average	SD	Minimum	Maximum
Motility (%)	110	81.1	8.0	47.8	92.2
Progressive motility (%)	110	64.4	10.5	31.2	84.6
Morphology normal (%)	110	78.1	8.5	52.7	93.0
Bent tail (%)	109	2.7	1.8	0.4	9.6
Coiled tail (%)	109	0.1	0.4	- 0.5	2.7
DMR (%)	109	4.0	2.9	0.9	17.1
Proximal droplets (%)	109	7.4	3.4	0.3	17.8
Distal droplets	109	6.0	2.9	0.7	14.9
Volume (mL)	109	349.4	104.4	184.0	672.1
Concentration (10^6^/mL)	108	152.7	46.4	60.1	301.1
Total concentration (billion)	101	46.4	19.1	8.0	102.8
Motility 96 h (%)	110	84.8	7.7	42.3	93.8
Progressive motility 96 h (%)	110	66.2	7.9	38.1	83.0

*p-values are shown in Supplementary Table 1 in Annex.

The CBVs for EC were highly correlated with each other (*p < 0.001*, Table 6). The number of grey pixels was positively correlated with DKL (*R*^2^ = 0.98) and negatively correlated with tubular density (*R*^2^ = −0.98), tubular area (*R*^2^ = −0.96), and diameter (*R*^2^ = −0.94). Similarly, DKL was negatively correlated with tubular density (*R*^2^ = −0.94), tubular area (*R*^2^ = −0.92), and tubular diameter (*R*^2^ = −0.89). Tubular density was positively correlated with tubular area (*R*^2^ = 0.99) and diameter (*R*^2^ = 0.98), and tubular area was positively correlated with tubular diameter (*R*^2^ = 0.98).

**Table 6:** Pearson’s correlation coefficients for the corrected boar values (CBVs) for echotexture traits in boars.

Echotexture traits*	Number of grey pixels	DKL	Tubular density (μm/cm^2^)	Tubular area (%)	Tubular diameter (μm)
Number of grey pixels		0.98	−0.98	−0.96	−0.94
DKL	0.98		−0.94	−0.92	−0.89
Tubular density (μm/cm^2^)	−0.98	−0.94		0.99	0.98
Tubular area (%)	−0.96	−0.92	0.99		0.98
Tubular diameter (μm)	−0.94	−0.89	0.98	0.98	

*All correlations were statistically significant (*p* < 0.05).

The correlation coefficients between the CBVs for EC and semen traits are shown in Table 7 (*p* values are in Supplementary Table 2 in Annex). The number of grey pixels and DKL were both negatively correlated with the TC (*R*^2^ = −0.51 and −0.54, respectively, *p* < 0.001), and with NM0h (*R*^2^ = −0.34 and −0.40, respectively, *p* < 0.0001). DKL was also negatively correlated with M0h (*R*^2^ = −0.19, *p* = 0.04), PM0h (*R*^2^ = −0.42, *p* < 0.0001) and M96h (*R*^2^ = −0.22, *p* = 0.02). On the other hand, tubular density, tubular area and tubular diameter were all positively correlated with TC (*R*^2^ = 0.51; 0.55 and 0.52; *p* < 0.0001), and NM0h (*R*^2^ = 0.32; 0.30 and 0.30, *p* = 0.001). Thus, young boars with higher DKL values have fewer sperm cells produced per ejaculate (billions) and those sperm cells will have a higher percentage of morphology abnormalities, and lower motility fresh and after storage. While the opposite is true for the higher values of tubular density, area and diameter.

**Table 7:** Pearson’s correlation coefficients for the corrected boar values (CBVs) for echotexture traits and semen traits in boars.

Traits*	Number of grey pixels	DKL	Tubular density (μm/cm^2^)	Tubular area (%)	Tubular diameter (μm)
Motility (%)	−0.13	−**0.19**	0.10	0.10	0.08
Progressivity (%)	−**0.36**	−**0.42**	**0.32**	**0.32**	**0.29**
Motility 96 h(%)	−0.18	−**0.22**	0.16	0.15	0.13
Progressivity 96 h (%)	0.03	0.01	−0.07	−0.09	−0.09
Ejaculate volume (mL)	−0.12	−0.14	0.10	0.12	0.08
Concentration (mill/mL)	−0.13	−0.17	0.12	0.10	0.13
Total concentration (billion)	**−0.51**	**−0.54**	**0.51**	**0.55**	**0.52**
Normal morphology (%)	**−0.34**	**−0.40**	**0.32**	**0.30**	**0.30**
DMR (%)	**0.23**	**0.25**	**−0.23**	**−0.24**	**−0.27**
Bent tail (%)	**0.42**	**0.47**	**−0.41**	**−0.41**	**−0.41**
Coiled tail (%)	**−0.26**	**−0.24**	**0.26**	**0.26**	**0.24**
Proximal droplet (%)	−0.12	−0.05	0.15	0.18	0.15
Distal droplet (%)	0.10	0.11	−0.08	−0.07	−0.08

*p-values are shown in Supplementary Table 2 in Annex. Highlighted values in bold indicate statistically significant correlation coefficients (*p* < 0.05).

## 4 Discussion

In this study we investigated the use of testicular ultrasonography and Computer-Assisted Image Analysis of testicular echotexture traits in young pre-pubertal boars as a tool to predict their future semen production and quality. Boars were subjected to testicular ultrasonography at 22, 24 and 26 weeks of age, during testing period at a production nucleus farm. At 27 weeks of age, these boars were transferred to two different semen production centers, where they began semen production and had several ejaculates collected and analyzed for a period of approximately 6 months. Corrected Boar Values were calculated for both, echotexture and semen traits, and correlations between those values were obtained. Higher values of DKL at pre-pubertal testicular ultrasonography were correlated with lower sperm motility and higher number of morphologically normal sperm cells per ejaculate. Since DKL values did not significantly change with age, this trait can already be measured in boars at 22 weeks of age, thus offering the possibility for early phenotypic classification and possible selection of boars. Early phenotyping of boars, and the possibility of early selection, would reduce the costs of semen production. The echotexture traits were highly correlated with each other, which might indicate a shared physiological background.

It is not surprising that while the number of grey pixels increased with age, that is, 22 to 26 weeks, the values of tubular density, area, and diameter decreased, showing a clear increase in testicular echogenicity during sexual development. An increase in testicular echogenicity with age from birth to puberty has been observed in several species, including humans (13), bulls (10, 12), and rams (14). However, this increase does not occur linearly, and rather numerous fluctuations occur, especially when more measurements are taken sequentially (15). Indeed, in this study population, the pattern of change in testicular echogenicity with age varied among boars. For the six boars that had the highest number of grey pixels at 22 weeks (average of 135.4) the average at 26 weeks showed no increase and rather a slight decrease (132.6). On the other hand, for the six boars that had the lowest number of grey pixels at 22 weeks (average of 97.0), the average at 26 weeks showed a continuous increase for this trait with age (121.5). Thus, we can conclude that there are different patterns of change in testicular echogenicity with sexual development in boars; however, boars with higher grey pixel values at a younger age will likely continue to show higher values as they get older, and vice versa. It is unclear why DKL values did not increase with the increase of age, and the specific reasons for changes in testicular echogenicity traits during sexual development remain unknown (15), demanding further investigations in boars.

Boars with higher values of tubular density, area and diameter produced ejaculates with a higher number of sperm cells with fewer morphological abnormalities. Previous reports have shown that testicular echotexture traits show a distinct temporal trend and are correlated with several histomorphological and endocrine attributes throughout sexual development (10, 15). The higher sperm production observed in boars with higher tubular density, area and diameter could have its physiological background in the histomorphological changes associated with the cellular proliferation occurring in the testicular parenchyma in boars during sexual development. The length and diameter of the seminiferous tubules are determined by specific windows of cellular proliferation and growth, leading to an increase in testicular size and to the start of testicular steroidogenesis and sperm production in boars (16, 17). From 12 to 16 weeks of age, boar’s growth in seminiferous tubules length is mainly related to the proliferation of Sertoli cells, which leads the way to the proliferation of germ cells (i.e., spermatogonia’s) from 16 to 20 weeks of age. It is a known fact that the higher the number of Sertoli cells, the higher the number of germ cells present in the testicles [*R* = 0.95, *p* < 0.05, (16)], and since each Sertoli cell can nurture up to 256 germ cells into sperm cells during a spermatogenic cycle, more Sertoli cells culminate in higher sperm production in boars (31). The proliferation of germ cells leads to further growth in the seminiferous tubule’s length and diameter, followed by a surge in Leydig cell proliferation in the testicular parenchyma interstitium, that triggers the start of steroidogenesis and a surge in testosterone levels at 19 weeks, starting spermatogenesis and marking the onset of puberty (16). So, it is the proliferation in the number of Sertoli, germ cells and Leydig cells that leads to an increase in tubular length and diameter, and to an increase in the cellular density in the testicular parenchyma, consequently increasing testicular size during sexual development, and finally the total sperm production capacity of the testicles (1, 5, 18, 19). Testicular size was not measured in this study, but it is very likely that boars with higher tubular density, area, and diameter also had larger testicles. For example, in this study population, left testicles had significantly higher values of tubular density, area and diameter and lower values of grey pixels and DKL, in comparison with the right testicles. And, in boars, left testicles are reported to be larger than the right testicles (8, 36, 37).

Larger testicular size is a good predictor of higher sperm production (20), but it is difficult to measure in live boars with their non-pendular testicles. Currently, reliable measurements of testicular size in boars can only be done with the use of B-mode ultrasonography, done by trained and experienced veterinarians. On the other hand, assessment of testicular tubular density, area and diameter with the use of echotexture analysis software is fast, objective, technician-independent and can be done in pre-pubertal boars. Studies on heritability of male traits in pigs have demonstrated that parameters related to testicular size, such as width, length, weight and volume; have high heritability [ranging from 0.33 to 0.44; (21, 22)]. So, echotexture traits could also have potential for genetic selection, although heritability and repeatability estimates are missing for these traits. Future research should be done to investigate the genetic background of echotexture traits and its usability for genetic selection programs.

The negative correlation between the number of grey pixels and DKL with total sperm concentration is probably related to the strong negative correlation between these two traits and tubular density, area and diameter, suggesting a lower presence of active seminiferous epithelium in these boars. Brito et al. (10) also found a negative correlation between testicular echogenicity index (i.e., higher values of grey pixels and white pixels) and seminiferous tubules area and diameter in bulls, although in bulls, higher testicular echogenicity was associated with higher daily sperm production. Also, stallions with compromised sperm production and quality due to testicular inefficiency had lower tubular area and density in comparison with those of stallions in the control group with normal spermatogenesis (11).

Boars with higher values of tubular density, area and diameter also had higher percentage of sperm cells with progressive motility and normal morphology, the latest mainly related to a reduction in the percentage of sperm cells with DMR and bent tails. Sperm cells acquire motility and progressive motility capacity during post-testicular sperm maturation that happens throughout their transit through the epididymis (34), and since epididymal development follows testicular development (23, 24), it is very likely that in these boars, epididymal function is also favored. There is a well-established paracrine regulation of structures within the testicles, with a strong significant positive correlation between the total number of Sertoli, Leydig and germ cells per testis, as well as between follicle stimulating hormone (FSH) and testosterone levels in boars. High testosterone levels within the testicles is known to improve epididymal function and favor post-testicular sperm maturation.

It is possible that an improved epididymal function also explains the higher percentage of sperm cells with normal morphology, lower incidence of DMR, and bent tails in boars with higher tubular density, area and diameter, as post-testicular sperm maturation also includes changes in sperm morphology. It is noteworthy that the migration of the cytoplasmatic droplet to the lower annulus of the sperm is a pre-requisite for normal sperm motility. Distal droplets remain attached to the sperm tail while within the epididymis, but they are released at the time of ejaculation (35), once the sperm cells come into contact with the seminal plasma (25). When looking into sperm cells within the epididymis, it is well known that most sperm cells within the cauda of the epididymis still have the distal droplet (33). The DMR (i.e., distal midpiece reflex) abnormality is the folding of the sperm distal midpiece of the tail before the release of the distal droplet, which is retained in the folding of the tail, thus indicating that this abnormality is caused by dysfunctions in the epididymis. In general, sperm cells with morphological defects, especially those on the sperm tail, do not show progressive motility during CASA analysis. Progressive motility has a negative genetic correlation with morphological abnormalities (26, 27). In fact, Gruhot et al. (26) reported a negative genetic correlation between progressive motility and DMR (*r*_g_ = −0.27) and bent tail (*r*_g_ = −0.24). Since DMR and bent tail are also moderately genetically correlated (*r*_g_ = 0.56), we can conclude that genes regulating biological pathways that lead to progressive motility are also regulating pathways that influence the occurrence of tail abnormalities in boars.

Finally, it is important to note that testicular echotexture and semen traits were measured in boars over an 8-month period in South Africa and were consequently subject to seasonal influences. In this study, tubular density, area and diameter increased from mid-winter in July to summer, peaking in January. On the contrary, the number of grey pixels and DKL decreased from winter (June) to summer (January). The total number of sperm cells per ejaculate followed the trend of tubular density, area and diameter and increased constantly from winter to fall, peaking in March, when the last ejaculates were collected for this study. Given the ages of the boars during this study, it is possible that sexual and testicular maturation have had a minor influence on the results regarding seasonality. However, others have reported that boars exhibit higher sperm production (28, 29) and testis size (29) during fall winter months. Further investigations regarding echotexture traits in young boars must include animals also in the northern hemisphere, and ideally for longer period of time covering all seasons of the year.

## 5 Conclusion

This study is, to the author’s knowledge, the first time testicular echotexture traits were measured in boars at 22 weeks of age and related to semen production. All echogenicity traits changed from 22 to 26 weeks of age in boars, except for DKL, indicating that: 1. the Ecotext software can detect changes in testicular parenchyma related with testicular development in boars during sexual maturation, and 2. that DKL can be measured in boars at 22 weeks of age making it possible to identify boars with high or low future sperm production capacity. An important added value of such traits is their correlation with semen quality traits, especially motility and morphology. Future research must be done on the potential use of these traits to objectively determine puberty onset in young boars.

## Data Availability

The raw data supporting the conclusions of this article will be made available by the authors, without undue reservation.
