# Supplementary material for: Assessment of testicular echotexture in young boars and its correlation with future semen production
Source: Front Vet Sci. 2026 May 6;13:1828393. doi: 10.3389/fvets.2026.1828393 (PMC13188863; doi:10.3389/fvets.2026.1828393)
Supplement: Supplementary file 1 [file Supplementary_file_1.pdf]

Supplementary Material

1 Supplementary Tables

Supplementary Table 1: p values for the Corrected Boar Values (CBVs) for semen traits in boars

**Semen Traits**	**Line(boar)**	**Collection Interval (d)**	**Age collection (months)**	**Season (month)**
Motility (%)	<.0001	0.11	0.32	0.43
Progressive motility (%)	<.0001	0.58	**0.0012**	0.34
Morphology Normal (%)	<.0001	0.32	0.29	**0.0006**
Bent Tail (%)	<.0001	**0.01**	0.11	**0.0003**
Coiled Tail (%)	<.0001	**0.01**	0.18	0.18
DMR (%)	<.0001	0.11	0.88	**<.0001**
Proximal Droplets (%)	<.0001	**0.05**	**0.01**	**<.0001**
Distal Droplets (%)	<.0001	**0.001**	0.56	**<.0001**
Volume (mL)	<.0001	**<.0001**	**0.0006**	**0.02**
Concentration (10^6/mL)	<.0001	**<.0001**	0.86	**0.03**
Total concentration (billion)	<.0001	**<.0001**	0.8	**<.0001**
Motility 96h(%)	<.0001	0.77	0.79	**0.0013**
Progressive motility 96h (%)	<.0001	0.73	0.34	**<.0001**

Supplementary Table 2: p -values Pearson's correlation coefficients for the Corrected Boar Values (CBVs) for echotexture traits and semen traits in boars.

**Traits***	**Number of Grey Pixels**	**DKL**	**Tubular Density (µm/cm2)**	**Tubular area (%)**	**Tubular Diameter (µm)**
Motility (%)	0.16	**0.04**	0.31	0.30	0.40
Progressivity (%)	**0.0001**	**<.0001**	**0.001**	**0.001**	**0.002**
Motility 96h(%)	0.06	**0.02**	0.10	0.12	0.16
Progressivity 96h (%)	0.78	0.94	0.49	0.36	0.34
Ejaculate volume (mL)	0.21	0.14	0.31	0.20	0.41
Concentration (mill/mL)	0.18	0.08	0.22	0.30	0.17
Total concentration (billion)	**<.0001**	**<.0001**	**<.0001**	**<.0001**	**<.0001**
Normal morphology (%)	**0.0001**	**<.0001**	**0.001**	**0.001**	**0.001**
DMR (%)	**0.02**	**0.01**	**0.02**	**0.01**	**0.005**
Bent Tail (%)	**<.0001**	**<.0001**	**<.0001**	**<.0001**	**<.0001**
Coiled Tail (%)	**0.006**	**0.01**	**0.006**	**0.01**	**0.01**
Proximal Droplet (%)	0.23	0.58	0.11	0.05	0.11
Distal Droplet (%)	0.29	0.27	0.39	0.47	0.42
